# Potential of rainwater harvesting in the retail sector: a case study in Portugal

**DOI:** 10.1007/s11356-023-25137-y

**Published:** 2023-01-17

**Authors:** Ana Ferreira, Vitor Sousa, Manuel Pinheiro, Inês Meireles, Cristina Matos Silva, Jorge Brito, Ricardo Mateus

**Affiliations:** 1 CERIS, IST, University of Lisbon, Av. Rovisco Pais, 1, 1049-001 Lisbon, Portugal; 2 RISCO, University of Aveiro, Campus Universitario de Santiago, 3810-193 Aveiro, Portugal; 3 ISISE – School of Engineering, University of Minho, 4800-058 Guimarães, Portugal

**Keywords:** Rainwater harvesting (RWH) systems, Retail store, Water stress, Water savings, Water footprint, Water intensity

## Abstract

Water is a crucial resource for life, and it is increasingly scarce in many regions of the globe. In addition, retail water use is responsible for up to 19% of public water globally supplied. Hence, this study has set out to explore the technical and economic feasibility of rainwater harvesting systems as an alternative water source for a retail store located in southern Portugal. Water consumption data from 2018 to 2021 was collected from water bills, placing average monthly water consumption at around 400 m^3^. Next, rainfall data was collected from the nearest meteorological station, comprising 54 years of daily rainfall data between 1932 and 2008 with an annual average of 685 mm. The simulation of a rainwater harvesting system was performed, resorting to the mass-balance model. The optimal tank size was found to be 100 m^3^ considering simply the relation with the relative water savings variation on the graph relating the water savings with the tank size. Results show that the simulated rainwater harvesting system would allow saving 32–36% of the water consumed, despite the store’s location in a dry climate, representing a financial gain of €330–372 per month. Findings suggest a substantial potential for the technical and economic feasibility of rainwater systems in retail stores, which makes them relevant solutions to achieve important water-savings in the retail sector, thus positively influencing retailers’ direct water footprint.

## Introduction

The global pressure on potable water is increasing due to population growth, poor land use management, pollution of water bodies, and climate change (Symeonidou and Vagiona 2018). Water scarcity is an important issue in many parts of the world. According to the United Nations (FAO and UN-Water 2021; UN-Water 2021), 2.3 billion people live in water-stressed countries, of which 733 million live in high and critically water-stressed countries. Even if most of these countries are in northern Africa and southern Asia, the area and population afflicted by droughts in the European Union (EU) augmented by almost 20% over the last 30 years. The situation is more expressive in Southern Europe, but is also increasingly present in countries such as the UK or Germany (European Parliament 2012). According to the Aqueduct Projected Water Stress Country Rankings (World Resources Institute 2015), Mediterranean countries are at high risk (level 3). High-risk water stress means that total annual water withdrawals (municipal, industrial, and agricultural) represent 40 to 80% of the total annual available blue water. In Portugal, in particular, water stress predictions for 2030 under a business-as-usual scenario are of high risk across the domestic, industrial, and agricultural sectors, with a tendency to grow in 2040.

Therefore, water is a threatened natural resource and improving water management, especially in countries with higher water stress levels, is of the utmost importance (UN-Habitat 2005). As such, water management in economic sectors with high water intensities, such as retail, is critical. It is estimated that water use in commercial buildings accounts for up to 20% of water withdrawal (US Environmental Protection Agency 2009; US Energy Information Administration 2012). Moreover, the mean water intensity of retail stores was found to be at 1123 L/m^2^ per year, and 47 m^3^ per worker per year (Ferreira et al. 2022). Water consumption in retail buildings could be reduced through more sustainability-driven management solutions. International regulations, such as the Water Framework Directive (European Parliament 2000) or the European Communication on Water Scarcity and Droughts (European Parliament 2012), are urging retailers toward sustainable water practice. Likewise, voluntary standards followed by top revenue retailers, such as goal 6 of the United Nations' Sustainable Development Goals, propose water access and sanitation for all people (United Nations 2015).

Improved water use in retail not only addresses political and social concerns but also enhances operational costs and environmental performance. In addition, the reduction of potable water consumption reduces greenhouse gas emissions associated with the water industry, distribution capture, and operation expenditures at a national level. Also, becoming water positive or closing the water cycle is a publicly expressed goal of some top revenue retailers (Coop Group 2015; ICA Gruppen 2015; Kohl’s_Corporation 2015; Lidl 2015; Norgesgruppen 2015; Walmart 2015; Kingfisher 2016; IKEA 2018), which aim firstly at reducing their water consumption and secondly their supply chain’s (ENDS Carbon - University of Edinburgh Business School 2009). To address water management issues in retail stores, top revenue retailers typically resort to two main categories of solutions: i) the reduction of water consumption mainly through water efficiency fixtures and ii) the identification of new water sources, mainly through rainwater harvesting (RWH) systems (BREEAM 2021). This study will focus in a RWH system applicable to a retail store in Southern Europe. A background of existing studies on RWH systems will be explored in the flowing subsections, as well as of water consumption patterns in retail stores. Water-saving solution currently used by retailers, which include RWH systems, will also be briefly disclosed.

### Background

#### Literature review

In general, a rainwater-harvesting RWH system comprises the collection, treatment, storage, and use of rainwater as a main or secondary water source (Silva et al. 2015). The collection of water usually takes place in roofs and terraces, which impact rainwater runoff quality and quantity. Next, the collected rainwater undergoes treatment, typically via a first flush device and a filtration device, and is stored in a tank, from which it is distributed to the projected end use point (Silva et al. 2015).

The literature review on RWH water-savings potential encompassed studies on multi-unit residential buildings and in arid regions of Australia (Eroksuz and Rahman 2010; Hajani and Rahman 2014), single and multi-family buildings in Spain (Domènech and Saurí 2011), and single-family buildings in Portugal (Silva et al. 2015). Other studies identified RWH water-savings potential for specific end uses (Basinger et al. 2010) and no particular building configuration (Palla et al. 2011, 2012; Campisano and Modica 2012; Mun and Han 2012; Rahman et al. 2012; Rashidi Mehrabadi et al. 2013). According to these studies, RWH-savings potential is context dependent. To obtain more robust results, a daily simulation of the RWH, which considers local climate and rainfall, water consumption pattern per end use, and RWH system configuration, is needed (Ghisi et al. 2007; Campisano and Modica 2014; Imteaz et al. 2015; Silva and Ghisi 2016). As such, to assess the feasibility of a RWH system in a retail store in southern Europe, a case study should be investigated, which is the aim of this research.

In European countries, namely, France, Germany, or Belgium, and in Japan, New Zealand, and the USA, RWH system use is being mostly for non-potable water in toilet flushing, washing, and irrigation (Herrmann and Schmida 2000; Schets et al. 2010). A comprehensive literature review on RWH system research can be found in Silva et al. (2017), which has found limited studies reporting on the efficacy of the performance of existing RWH systems, alike Zaizen et al. (2000) and Ward et al. (2010).

RWH systems have received relatively short research interest in Portugal: a study by Oliveira (2008) assessed the economic feasibility of RWH systems using 10-year rainfall data. Amado and Barroso (2013) assessed the feasibility of RWH systems in residential buildings, predicting water savings of 43.2% and 31.5% for single-family buildings and multi-family buildings, respectively.

There are, however, very few studies regarding RWH systems in commercial buildings, even though in these buildings, the proportion of water that does not need to be potable may represent up to 75% of total water consumption (Proenca and Ghisi 2010). Studies regarding RWH systems in commercial buildings included the evaluation of alternative water sources for commercial buildings in Australia (Cook et al. 2014), the design of a wall-mounted rainwater harvesting system faced with limited space (Foo et al. 2017), the cost comparison of new or retrofitted RWH systems in commercial buildings (Lani et al. 2019), the performance of small- and large-scale RWH systems in commercial buildings in Malaysia according to future water tariffs (Lani et al. 2018), the life cycle assessment of a RWH system (Ghimire et al. 2017), and alternative water sources in New Zealand’s commercial buildings (Bint et al. 2019). Other authors studied tank sizing and conducted the economic analysis of a rainwater-harvesting system in a commercial building (Matos et al. 2013a, 2015).

Out of the studies mentioned, only three addressed RWH systems in retail buildings in the regions of Malaysia and New Zealand (Lani et al. 2018, 2019; Bint et al. 2019), and an additional one analyzed case studies in Portugal and Brazil (Sousa et al. 2019). Hence, this study is novel in assessing the feasibility of a RWH system in a retail store in a Mediterranean climate, where the water stress level is higher, and the rainwater frequency tends to be lower. To the best of the authors’ knowledge, no other studies examine the economic feasibility of RWH systems in retail buildings, which is a gap in knowledge this study will address.

#### Water use in the retail sector—water consumption pattern

Assessing water consumption indicators in retail is not straightforward. Water consumption patterns may vary according to the sector’s diversity (Morales and Heaney 2014), namely, store type, customer frequency, sales area, number of workers, installed equipment, and landscaping. In a water intensity indicators’ study (Ferreira et al. 2022), specific water use metrics for retail buildings were identified, which included the water intensity per store area (WIA), water intensity per store (WIS), water intensity per worker (WIW), or water intensity per revenue (WIR). This study has placed mean WIA values per store area at 1123 l/m^2^/year; mean WIS values at 4411 m^3^/store/year; mean WIW values at 47 m^3^/worker/year; and mean WIR values at 483 l/billion$/year.

For non-food retailers, such as in the case of the present case study, the number of workers was a reliable predictor of water consumption. Statistically significant differences in the mean confirmed that WIA varied according to the continent where the stores were located, and WIS varied according to the dominant operational size of the stores. In a unidimensional analysis, WIS had a statistically significant correlation with the number of stores, the average store area, and the total stores. In addition, WIW and WIR were correlated with store area. Store type was statistically significant in a multidimensional analysis when controlling for the continent in the WIW indicator. Additionally, ordinary least square (OLS) methods showed that the continent and the stores had a statistically significant influence on WIA.

Food retailers seem to have a higher mean of WIA when compared to non-food retailers, which may be explained by display and preparation requirements in food departments, as well as icemakers (US Environmental Protection Agency 2009). Also, floor sanitation in food preparation and receiving areas is more water intensive than non-food retail. In contrast, non-food retailers seem to use water primarily for restrooms, space cooling (Gleick et al. 2003), and landscape irrigation.

Other general differences in retailers’ water performance may be related to merchandising, store occupancy, water efficiency and choice of water fixtures, occupant’s behavior, and maintenance. Marketing and client segmentation strategies may also influence water consumption in heating and cooling systems, restrooms, and landscape irrigation. Local culture could also impact the variability of water intensity since most of the lowest water intensity values were found in retailers in European countries, followed by American, and lastly by Asian countries. In a RWH system study, it is important to characterize the water consumption of the retail store to estimate the percentage of savings that can be expected and determine the feasibility of the system.

#### Water-saving solutions in retail

RWH systems are one of the water-saving solutions most cited by retailers. Overall, the most reported water-saving solutions by top revenue retailers are high-efficiency fixtures (48%), water management systems (48%), and rainwater harvesting (41%) (Ferreira et al. 2019). Working with non-governmental organizations in water programs also ranked high among retailers (41%), which reinforces water as an important corporate social responsibility issue. Retailers, in their sustainability reports, do not disclose the extent to which water-saving solutions are implemented in the stores. While high-efficiency fixtures may be more generalized for their low cost and ease of installation, RWH systems, for instance, may be restricted to a few stores. Regarding landscaping, the use of indigenous plants (22%) and smart irrigation systems (15%) were the most cited solutions by top revenue retailers. Cleaning systems were not frequently cited by top revenue retailers, though optimizing cleaning-in-place processes, reusing rinse water to clean truck tanks, and dry ice blasting were mentioned by 4% of them (Ferreira et al. 2019). As for water management solutions, water sub-metering (15%) and leak repair (11%) were the most cited measures by retailers. Additionally, water consumption reduction could be hampered by behavior and maintenance factors, highlighting the importance of human demeanor in water reduction strategies. Staff training on water savings was mentioned by 15% of retailers, whereas selling water sensitive products to clients scored 22%. Lastly, 4% of retailers mentioned the need of developing a water manual for suppliers as a mean to address the supply chain’s indirect water footprint (Ferreira et al. 2019).

Cost reduction and the minimization of water risks seem to be corporate driving factors in retail toward water stewardship (Best Buy 2015; Costco 2015; Walmart 2015; E. Leclerc 2016; Wesfarmers 2016; Auchan 2017; Rewe 2017; Target 2017; Sainsbury plc 2018; Woolworths 2018). Nonetheless, studies estimate that the financial payback of water-efficiency strategies, namely, that of RWH systems, is context dependent, despite the environmental benefits of water savings (Sousa et al. 2019). Cost-effective RWH systems could include the pretreatment of rainwater to be reused safely onsite, particularly on toilets, flushers, cleaning, and landscaping (namely, in smart irrigation systems, such as drip irrigation), in turn reducing water demand during drought or in water-stressed regions in new or renovated retail stores.

Alongside legal requirements, investment cost, management beliefs, and treatment requirements have been some of the most important barriers hindering the application of RWH systems in retail stores. On the contrary, water scarcity issues, corporate social responsibility concerns, and operational costs of domestic water have been pushing retailers to try different solutions to address natural resource efficiency. Hence, the following case study has been developed to support decision-making regarding the technical and economic feasibility of a RWH system in a Portuguese retail store, in a Mediterranean climate.

### Research goals

Given the lack of studies of RWH systems in retail buildings, in general, and in Mediterranean climates, in particular, this paper aims to assess the technical and economic potentials of a RWH system in a large standalone retail store in southern Portugal, owned by a top revenue global non-food retailer. This store is characterized by a large roof surface and by 70–90% water consumption in non-potable end uses, such as irrigation, cleaning services, carpark washing, and toilets. This is in line with the Portuguese decree-law 23/95 which authorizes non-potable water use exclusively for irrigation, pavement washing, firefighting, and other nonfood-related activities (Ministério das Obras Públicas 1995). Likewise, the Water and Waste Services Regulation Authority (ERSAR) guidelines on water use efficiency limit the use of harvested rainwater to non-potables uses, essentially irrigation (Almeida et al. 2006a).

In this study, two specific questions were investigated as research goals: i) “Are climate conditions in southern Portugal compatible with the implementation of RWH systems in retail stores?” and ii) “Is it economically feasible to install RWH systems in retail stores?*”* The present case study provides insight regarding the economic feasibility of RWH systems in retail stores as efficient alternative water sources, which is a contribution to the existing body of knowledge. Furthermore, lessons learned in the retail sector are easily disseminated among peers, thus fostering enhanced sustainable water management across the sector.

## Materials and methods

### Description

**Fig. 1:**
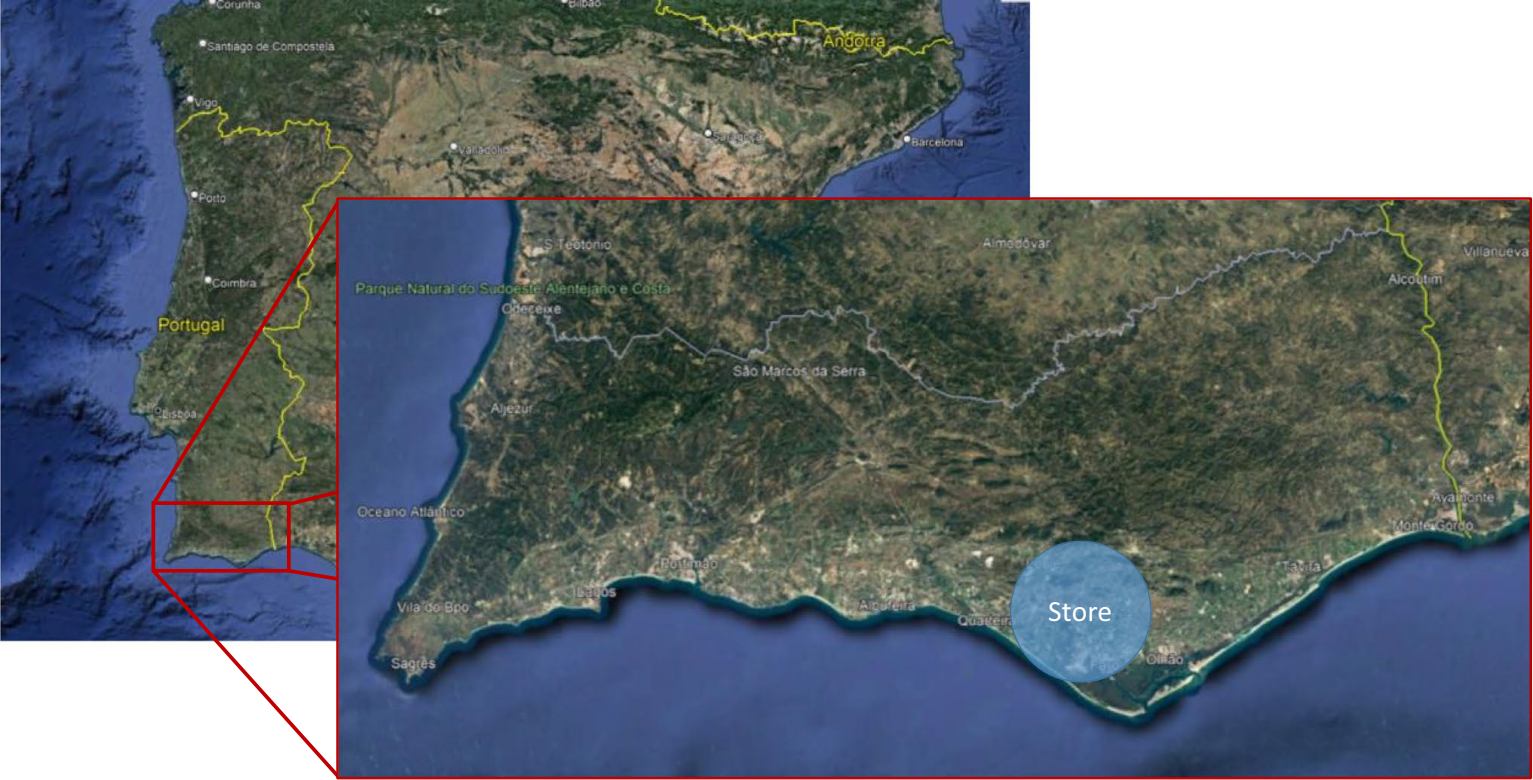
Approximate location of the retail store in Algarve, Portugal

A retail store located in the South of Portugal (Algarve region) was selected as a case study (Fig. 1). The store is part of one of the top global do-it-yourself (DIY) and home improvement retail groups, operating in Europe, South America, and China.

In addition to the availability of the data on water consumption at the store, this store was selected as a case study because it is one of the group’s most recent stores in Portugal (it opened to the public in 2017), with enough years in operation to collect data from. Therefore, the water consumption of the store reflects modern water consumption standards of the group (and of the sector) and water losses from leaking water installations should be residual or inexistent.

### Data collection and assessment

The data required were obtained from two sources: i) the retail store management and ii) the National Information Service on Water Resources.

**Fig. 2:**
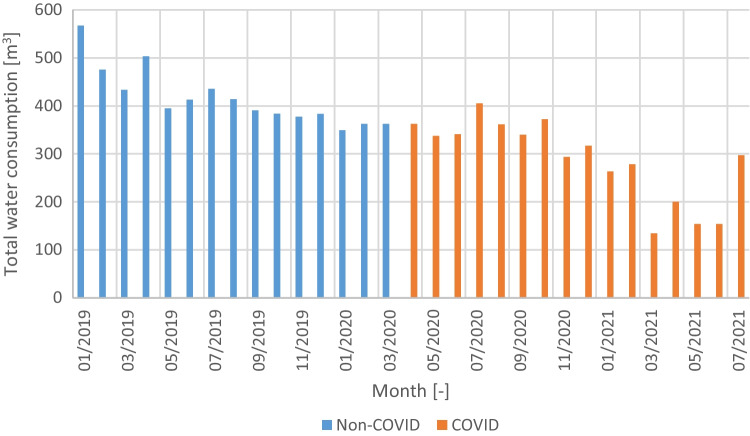
Total water consumption pattern from 2019 to 2021

The retail store management provided the monthly water bills from December 2018 to July 2021 (the last available month at the time of the data collection). This allowed the analysis of the water consumption pattern during a non-COVID period (until March 2019) and a COVID period (since April 2019) (Fig. 2).

Detailed measurements of the water consumption by end use in the store are not available, so expert insight from the retail store management was required to estimate the proportion of the total water consumed in non-potable uses (e.g., toilet flushing, cleanings, and heating, ventilation, and air—HVAC). According to the retail store management, the non-potable water consumption ranges between 70 and 90% of the total water consumption, with an average value of 85%. The expert estimates from the store management team are consistent with the end uses reported by Gleick et al. (2003), in which 70% of the water consumed in retail building is in exclusively non-potable end uses (HVAC, landscaping, and other uses), 30% in restrooms (26%) and kitchens (4%). Regarding the water consumption in the restrooms, in a retail store it is split only into toilet flushing and tap use. Considering the study of Almeida et al. (2006b) that analyzed the detailed water end use in 40 households in Portugal, it is possible to extrapolate that toilet flushing corresponds to 65% and tap use 35% of the water consumption in restrooms. Combining the information from both sources results in an estimated non-potable water consumption of 86.9%. This is a conservative estimate considering that this store does not have a kitchen and that the tap water consumption in residential building restrooms satisfies needs that are not present in retail buildings (e.g., shaving, brushing teeth). The expert estimates are also consistent with the non-potable water consumption of the largest shopping mall in Portugal. From the study of Sousa et al. (2019), the non-potable water consumption outside the stores (HVAC, fountains, cleaning, and landscaping) of the Colombo shopping center is 70.2%. In this mall, the separate metering of 4 restrooms using graywater in the toilets revealed that 90% of the water is used in the toilets and only 10% in the taps. This corresponds to a non-potable water fraction of 97%, indicating again that the expert estimate for the retail store under analysis is most probably conservative.

The roof has an area of 9315 m^2^, which is approximate to the net store floor area (the parking is under the store). This corresponds to specific water consumptions of 0.55 m^3^/m^2^, in 2019, and 0.45 m^3^/m^2^, in 2020. This is also consistent with the specific water consumption of the 15 retail stores reported by the US Energy Information Administration (US Energy Information Administration 2012), ranging from 0.34 to 1.01 m^3^/m^2^, with an average value of 0.58 m^3^/m^2^.

**Fig. 3:**
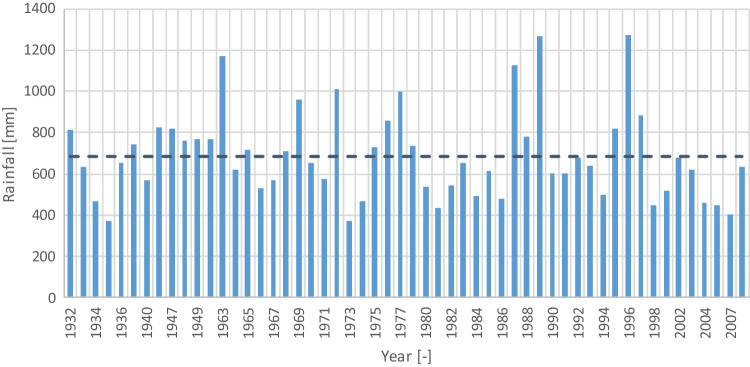
Annual rainfall pattern in mm from 1932 to 2007

The National Information Service on Water Resources (Agência Portuguesa do Ambiente 2021) provides historical weather records from more than 700 meteorological stations spread over Portugal. Analyzing the stations’ records closest to the store, the Loulé station (code 31I/01UG) presented the most extended and complete rainfall series, with 54 years of daily rainfall data between 1932 and 2008. The data from the most recent years is not available because the economic crisis that affected Portugal harshly compromised the maintenance of most stations and the quality check of the data. Loulé station is located roughly 7 km north of the retail store. The average annual rainfall is 685 mm, one of the lowest in Portugal, ranging from less than 400 to over 1200 mm (Fig. 3).

### Methodology

The approach adopted in the research entails two main components: i) the statistical analysis of the base data and ii) the simulation of a hypothetical RWH system.

The water consumption data and rainfall records were analyzed to identify the existence of trends, outliers, correlations, and statistically distinct groups. Trends were assessed for both water consumption and rainfall record resorting to the typical OLS regression. The *t*-test was applied to assess if the average water consumption was distinct before and during the COVID period. Outliers were identified in the rainfall record using the Tukey criterion, which is a non-parametric method and does not require the assumption of normality of methods such as the *Z*-score. Also, non-parametric Spearman and Kendall correlations were used to evaluate the existence of statistical correlations between the annual rainfall and the rainfall in each month and between the different months.

The simulation of a hypothetical RWH system was done, resorting to the typical mass-balance model. The model states that the mass entering the system must equal the mass leaving it plus the mass accumulation within the system, which corresponds simply to applying the mass continuity equation to a RWH system in each time step of the simulation. Herein, a daily time step was considered because it corresponds to the rainfall data’s resolution. Since the water consumption data has a monthly resolution and the store is open 7 days per week, uniform daily water consumption was considered in each month.

Linking the mass-balance model with the components of a RWH system and considering the tank as the central component, the mass accumulation is governed exclusively by the tank volume, while the water entering and leaving the system depends on the dynamic interaction of various factors. Herein, the tank volume varied between 20 and 1000 m^3^. A volume of 1000 m^3^ corresponds roughly to 3 months of water consumption of the retail store and was simulated only to capture the maximum potential rainwater use, since in practice a tank this size is not viable from a financial point of view and raises water quality problems due to the storage of the rainwater for a long period of time.

The water entering depends on the interaction between the rainfall pattern, the collection area, and the runoff coefficient. The rainfall pattern can be adequately characterized from historical rainfall records, assuming that the observed values in the past are an accurate representation of the future. This may not be the case under the climate changes context in many regions of the globe. Disregarding the potential influence of wind-driven rain, the collection area is accurately represented by the area of the horizontal projection of the roof from which the rainwater is drained to the tank. The runoff coefficient defines the fraction of the rainwater precipitating in the collection area flowing into the tank. Basically, it represents the water losses in the collection and is the most complex factor driving the water entering the system. In fact, it is the most complex factor driving the performance of a RWH system because it depends on the dynamic interaction between endogenous and exogenous building characteristics. The former are the characteristics of the roof, namely, slope and material, and the existence of first-flush devices. In theory, sloped roofs present higher runoff coefficients than flat roofs, and impervious, regular, non-porous materials (e.g., metal sheets) will have higher runoff coefficients than pervious, irregular, porous materials (e.g., green roof). The first-flush devices discharge the initial rainwater collected, which usually are the most polluted. The exogenous characteristics influence the evapotranspiration and surface retention, including aspects such as temperature, radiation, wind speed, and humidity. Furthermore, these endogenous and exogenous factors have a variable interaction over time. The best example is probably the case of a green roof, with the development level of the vegetation and the water content in the soil before each rainfall event varying throughout the year and affecting the evapotranspiration and surface retention. The first-flush devices may also be dynamic because some discharge a fixed volume of rainwater while others define the amount of rainwater discharged depending on some water quality parameter (e.g., turbidity). The collection area considered is the entire roof area, and two alternatives were considered to model the water losses: i) a runoff coefficient of 0.8 and ii) an initial water loss of 2 mm. In the first option, the rainfall in each day is simply multiplied by 0.8, which accounts for all water losses in the collection. In the second option, only the first 2 mm of a series of consecutive days with rain is lost, implying that the evapotranspiration is insignificant after this initial loss (surface retention and first flush). The first option is the most typical for residential buildings. Still, the second may be adequate to represent a typical retail store roof (metal sheets) and drainage system (siphon roof drainage).

In addition to the water losses in the collection, which represent water exiting the RWH system but not the tank, the water exits the RWH tank through consumption within the building and overflow. The monthly water consumption reported by the store management is assumed to be uniformly distributed over the days of the respective month. Still, there may be differences depending on the number of visitors in each day and the maintenance schedule. Overflow occurs whenever the amount of water collected exceeds the available storage capacity of the tank. This last aspect may be affected by the spillage algorithm adopted, namely, if the consumption occurs before or after the rainfall. This is a modeling simplification necessary to represent the order in which the demand for water and the supply of rainwater occurs in each time step of the simulation. In addition to the traditional demand before rain (DBR) and demand after rain (DAR), herein, a mixed option (MIX) was also considered, in which 50% of the rain precipitates before the consumption and 50% after. This third option is introduced since rainfall may occur before, after, and during the demand. The error introduced by this assumption decreases with the reduction of the time step and the increase of the water tank volume.

The option of considering annual continuity implicitly implies that a specific sequence of years in term of rainfall will occur in the future. Therefore, simulations were done considering that there is no inter-annual continuity, so the tank is considered empty at the beginning of each year. Three parameters were computed in all simulations: i) the total water savings, ii) the non-potable water savings, and iii) the rainwater wasted. The first two parameters reflect the percentage of the total and non-potable water consumption that is supplied with rainwater, respectively. The total water savings have a limit corresponding to the fraction of the water consumed in non-potable uses, while the non-potable water savings have a limit of 100%. The rainwater wasted is the ratio between rainwater discharged by overflow of the tank and the rainwater that precipitates over the roof. It has a limit of 100%, and the complementary indicates the amount of rainwater consumed for non-potable uses in the building plus the water losses in the collection.

The calculation process and the formulas are detailed in Figs. 9 and 10 of the Appendix.

## Results and discussion

### Water consumption and rainfall pattern analysis

The COVID pandemic affected the water consumption in the store, but with a time delay (Fig. 2). Therefore, the water consumption patterns in each period are statistically distinct according to the *t*-test results using bootstrapping to compensate for the small sample size and the variability between months regardless of the COVID (*t*(30) = 4.757, *p* < 0.01). The year 2018 was not included in the analysis because there is always a period of optimization of the operation and maintenance of any new store, still noticeable in the decreasing water consumption pattern at the beginning of 2019. However, between March 2019 and March 2020, the water consumption was relatively stable, ranging from 350 to 400 m^3^ in most months. Assuming that the water consumption pattern since the start of the COVID pandemic is abnormal, the analysis will be done using the water consumption records of 2019, which averaged 430 m^3^ per month.

Despite the inter-annual variability, there are no statistically significant increasing or decreasing trends in the total annual rainfall. However, the 3 years with the highest rainfall (1963, 1989, and 1996) are identified as outliers using the Tukey criterion.

**Fig. 4:**
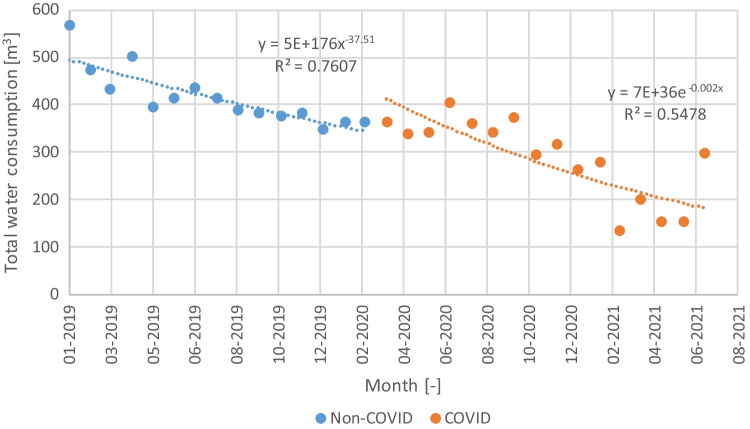
Total water consumption pattern functions from 2019 to 2021

With an average of 57 mm, the monthly rainfall pattern (Fig. 4) reveals marked wet (winter) and dry (summer) seasons, with smooth transitions in spring and autumn. The dispersion of the rainfall amount in each month is substantially higher, with several months having outliers based on the Tukey criterion (dots). These outliers indicate the existence of extremely wet months, in some cases recording over 300 mm.

Using the Spearman’s rho and Kendall’s tau correlations, because the rainfall distribution in each month is not normally distributed, it was found that there are statistically significant positive correlations between the rainfall in the months of November (Spearman: *r*(52) = 0.361, *p* < 0.01; Kendall: *r*(52) = 0.244, *p* < 0.01), December (Spearman: *r*(52) = 0.386, *p* < 0.01; Kendall: *r*(52) = 0.256, *p* < 0.01), January (Spearman: *r*(52) = 0.536, *p* < 0.001; Kendall: *r*(52) = 0.391, *p* < 0.001), and February (Spearman: *r*(52) = 0.320, Kendall: *p* < 0.05; *r*(52) = 0.215, *p* < 0.05) and the annual rainfall. This indicates that it is the variation of the rainfall in these wetted months that governs the variability of the total rainfall. This has implications on the expected performance of RWH systems since a wetter year does not necessarily imply a higher potential for rainwater use. The concentration of the extra rainfall in the wettest months is more probable to increase the volume of overflow because it is not viable to build a tank large enough to store the excess water to use in the dry months.

Between the various months, there is a statistically significant negative correlation between May versus July and September versus June for both Spearman’s rho and Kendall’s tau correlations. Based on the Kendall correlation alone, there is also a positive statistically significant correlation between January and February. This implies that the rainfall in each month may be regarded as independent in most cases since even the statistically significant results present very low correlation coefficients.

Similar patterns were found analyzing the rainfall pattern considering the hydrological year (October 1 to September 30) instead of the civil year (January 1 to December 31), but the correlations are stronger. This was expected since a wet October, November, and December is more likely to be related to a wetter January and February and January of the following year than in the same year. In terms of the annual rainfall, only the hydrological years of 1962–1963 and 1995–1996 are identified as outliers using the Tukey criterion.

### RWH system performance simulation

#### Influence of the tank size

**Fig. 5:**
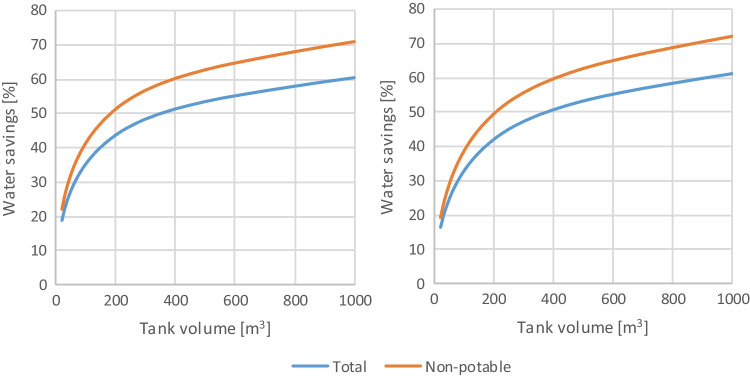
Water savings evolution in % and tank volume in m^3^ considering a constant runoff coefficient (left) and a constant initial water loss (right)

Figure 5 presents the typical evolution of the water savings with the tank volume. Since the non-potable water uses are assumed as a constant fraction of the total water consumption, the curves are parallel, and the conclusion drawn from analyzing one applies to the other. Unless specifically stated otherwise, the following results will report the total water consumption. The total water savings tends to 66%, considering a constant runoff coefficient, and 69%, assuming a constant initial water loss, when considering a tank with infinite volume. This means that the rainfall possible to capture in the store’s roof is not enough to supply the total water demand.

**Fig. 6:**
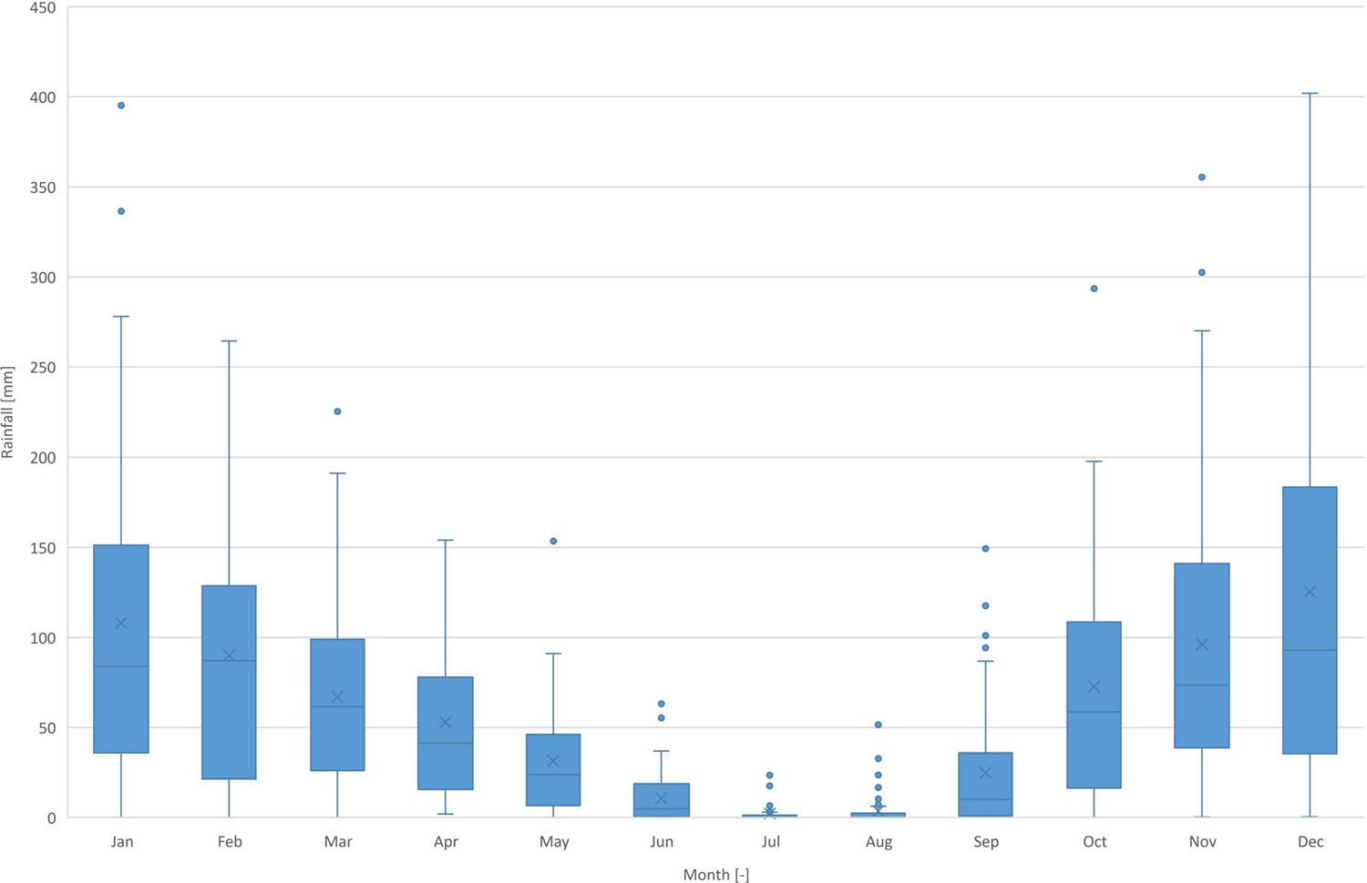
Boxplot of the monthly rainfall pattern in mm during a year, including outliers

The tank is the most expensive component of a RWH system without any water treatment. Both the tank and the rainwater distribution network are higher in an existing building than in a new building. Therefore, the transition between exponential and the asymptotic water savings growth in the tank volume versus water savings graph tends to correspond to the optimal volume from a financial point of view. In this case study, the transition takes place for a tank volume of approximately 100 m^3^, that is, the place in Fig. 6’s graphics where the growth rate of water savings starts to reduce significantly. Hence, a tank volume of approximately 100 m^3^ will be used as the reference for the following analysis.

#### Influence of the algorithms used

**Fig. 7:**
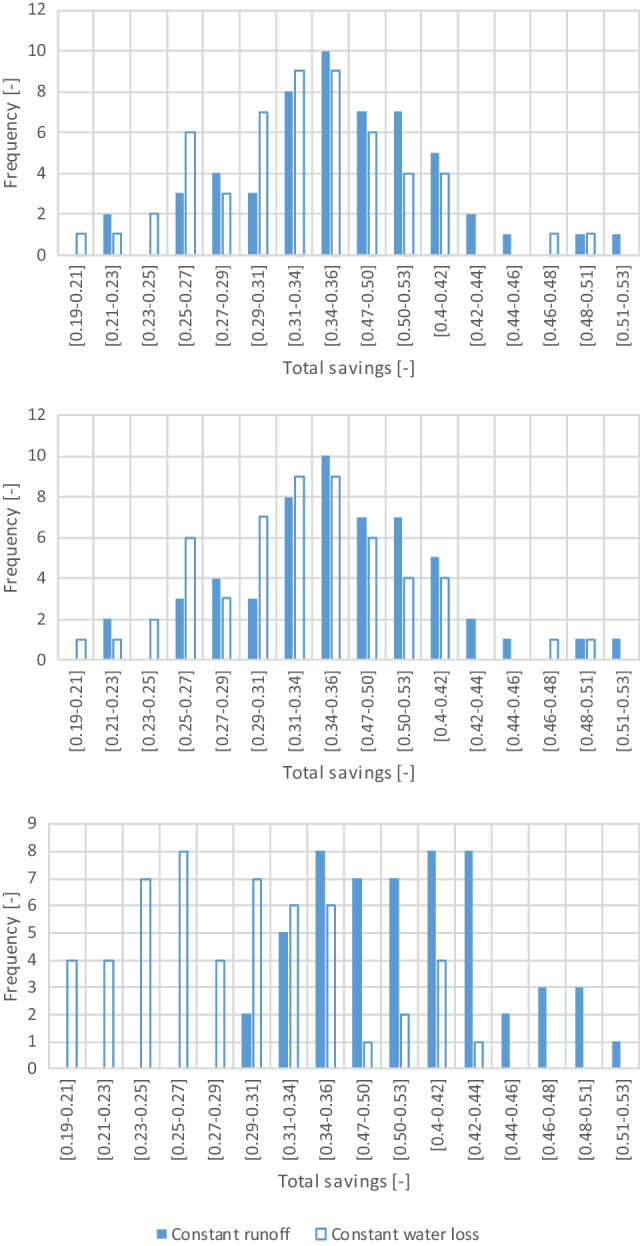
Histogram of the total water savings (top), non-potable water savings (middle), and rainwater use efficiency (bottom) for a tank volume of 100 m.^3^

The histograms of the annual total water savings and rainwater use efficiency for the simulations considering a constant runoff coefficient or a constant initial water loss and using the DBR spillage algorithm are depicted in Fig. 7. The histograms are similar regarding the total and non-potable water savings, with higher water savings being slightly more frequent on the constant runoff coefficient simulations. This is reflected in a 2% difference in the average water savings between the two approaches, which is consistent with the 3% difference found when comparing the case of a tank with infinite capacity. The difference is not statistically significant using the *t*-test, regardless of the spillage algorithm used (DAR, DBR, MIX). The histograms of the rainwater use efficiency are statistically different based on the *t*-test results (*t*(106) = 9.039, *p* < 0.001). The parametric *t*-test was used because the results considering a constant runoff coefficient and a constant initial water loss were found to be normally distributed using the Shapiro–Wilk test (*W* = 0.973, *p* > 0.05). Still, similar conclusions were obtained using the equivalent non-parametric Mann–Whitney *U* test (Mann–Whitney *U* = 330, n1 = *n*2 = 54, *p* < 0.001 two tailed).

**Fig. 8:**
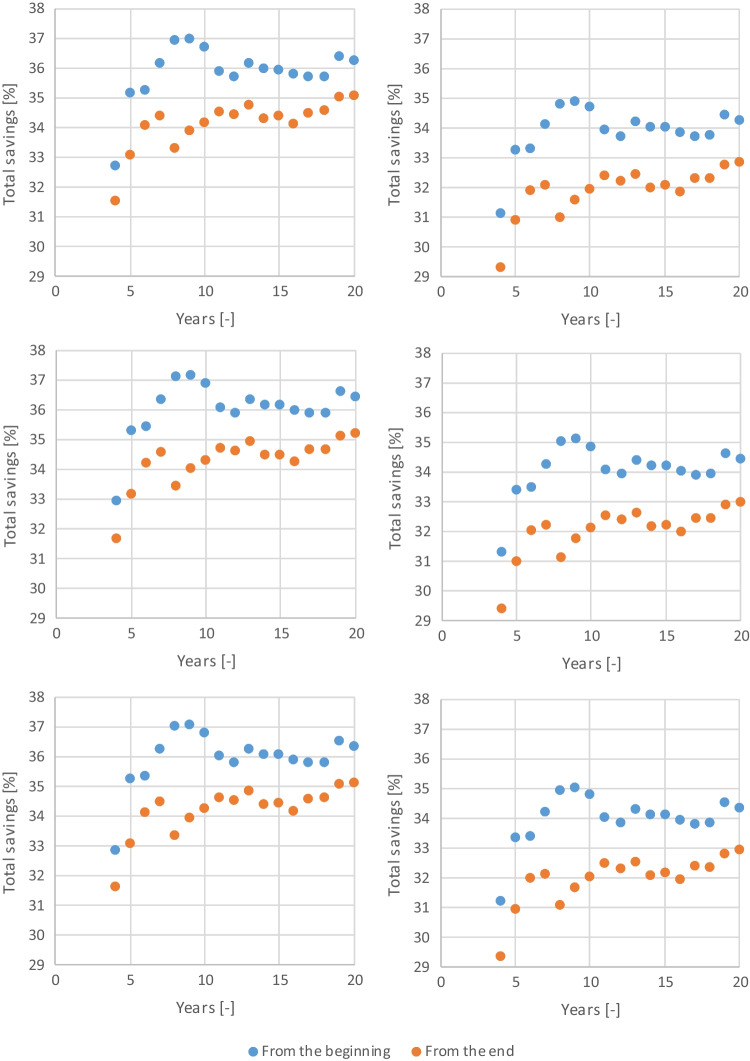
Total water savings using the DBR (top), DAR (middle), and MIX (bottom) algorithms, considering a constant runoff coefficient (left) and a constant initial water loss (right) for a tank volume of 100 m.^3^

**Fig. 9:**
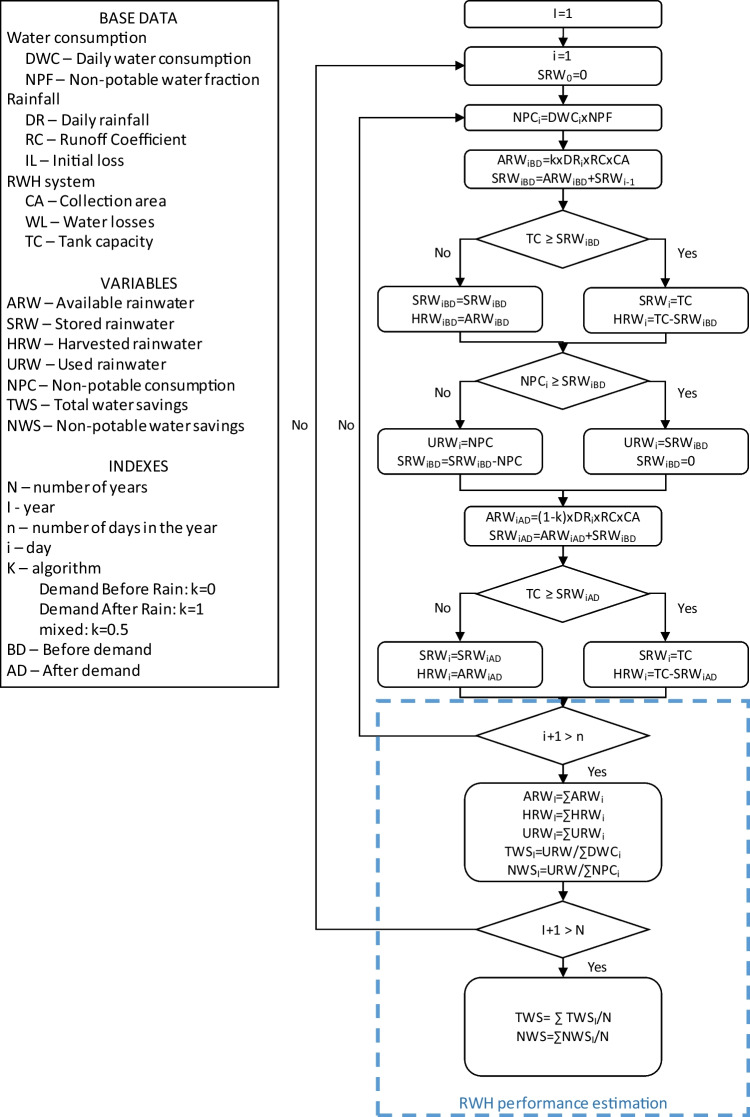
Rainwater harvesting simulation using a constant runoff coefficient to model the water losses

**Fig. 10:**
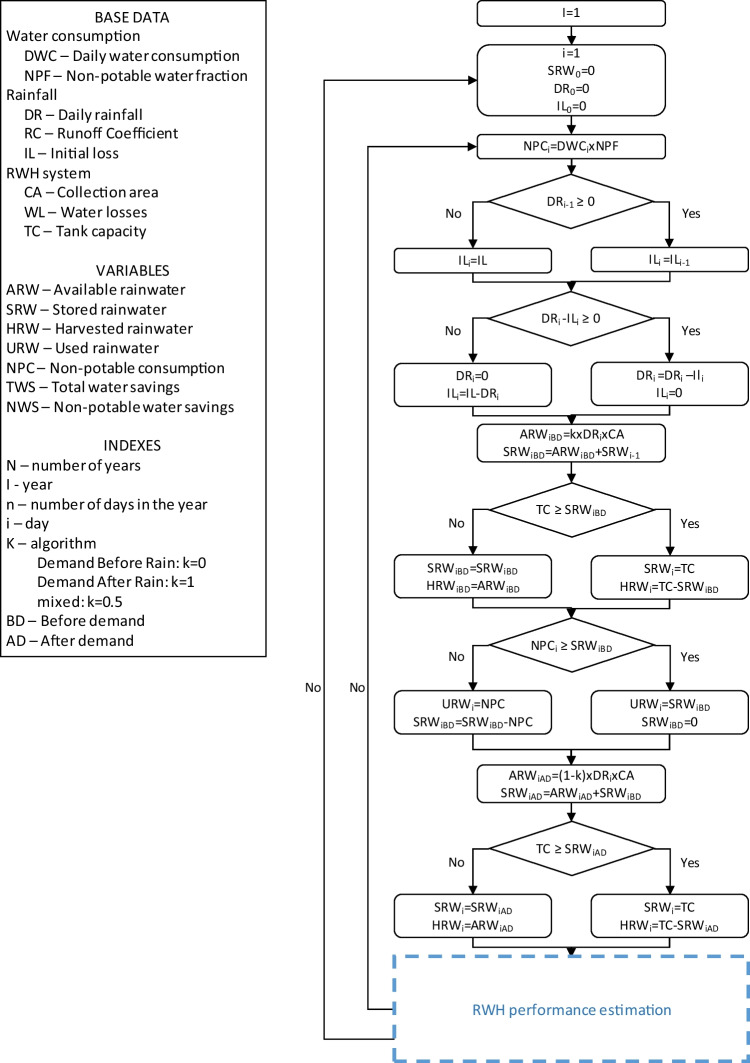
Rainwater harvesting simulation using a constant initial water loss to model the water losses

The influence of the spillage algorithm used for the total water savings was assessed along with the length and starting year of the rainfall series, as depicted in Fig. 8.

The spillage algorithm used was found to have a negligible effect on the results, which was expected since the tank volume is between 6 and 10 times the daily non-potable water consumption. For the retail store analyzed, the rainfall pattern and the roof area create conditions for the results to be independent of the spillage algorithm used regardless of the tank volume. The rainfall data, however, is found to influence the results. Using the first years of the series yields higher water savings than the last years of the series. This is explained by differences in the rainfall patterns, with the first years being wetter than the previous years of the series. The average annual rainfall of the first 20 years of the series is 706 mm, not much higher than the 692 mm of the last 20 years. However, the average is not a robust measure of central tendency. When comparing the medians, the difference becomes noticeable, with the first 20 years having a median of 715 mm while the last 20 years only 630 mm. These results highlight the impact of the rainfall variability at an annual scale on the performance of the RWH system. In this case study, the water savings decrease roughly 1% when the annual rainfall pattern changes from relatively homogeneous to being characterized by a more marked alternation of dry and wet years. The length of the rainfall series used has the most significant impact in the results, with a 4% difference between using a series of 4 or 20 years long. Considering that the water savings using the full rainfall series is 35%, using a constant runoff coefficient, and 33%, using a constant initial water loss, selecting at least 11 years for simulating the RWH system would result in a difference of less than 1.5% in terms of water savings regardless of using the first or last years of the series.

### Financial value of the water savings

Despite being in one of the driest regions of Portugal, a RWH system with a tank of 100 m^3^ would allow saving roughly a third of the total water consumed in the retail store studied. Assuming an average monthly consumption of 400 m^3^, this corresponds to direct financial savings of around €360 per month. This is because water costs are relatively low (€1.07/m^3^, plus a fixed fee of €2.68), but the sanitation and solid waste costs are indexed to the amount of water consumed from the public network, totaling almost €2.5/m^3^. An accurate estimate of the investment cost associated with the RWH system is not available, since the cost of installing the rainwater distribution network in a store in operation is not straightforward. Furthermore, the characteristics of the pumping system required could only be defined by analyzing the water infrastructure of the store in detail (number, flowrate, and location of the water points to determine the peak discharge and the minimum pressure). Nevertheless, results show a potential to reduce water consumption in the retail sector by implementing RWH systems. In addition, increasing populations, urbanization, and climate change will push the rising demand for alternative water management options, as pointed out by Bint et al. (2019).

Unfortunately, there is no measurement of the water end use. This is a limitation since the non-potable water fraction had to be estimated based on expert opinion from the store management. Nevertheless, this is limitation common to most of the studies on RWH. In fact, some studies on commercial buildings in Portugal did not even have the total water consumption data (Matos et al. 2013b) and assume constant monthly water consumption estimated from typical capitations. Still, varying the fraction of non-potable water between 70 and 90% the water savings ranges between 32 and 36%, respectively. The corresponding monthly savings in terms of the water bill vary between 330 and €372, which should be a big impact on the financial viability of a RWH system.

## Conclusions

This study examined whether Mediterranean climate conditions in southern Portugal could be compatible with the implementation of RWH systems in retail stores, with a positive result. Despite the store’s location in Algarve, one of Portugal’ driest areas, a RWH system with a tank of 100 m^3^ would allow saving approximately one-third of the total water consumed in this store. Nevertheless, rainfall data influence water-saving results and, for the case study analyzed, selecting at least 11 years as a baseline for simulating the RWH system would produce more robust results, despite the choice of years selected from Loulé meteorological station’s rainfall series. Still, with 6 or more years, the results start to become stable. Results are thus encouraging in analyzing RWH systems’ technical feasibility in other regions, where rainfall is more abundant.

The findings suggest a high potential for the technical and economic feasibility of RWH systems in retail stores, contrary to popular management belief that presumes RWH systems as cost ineffective. The major limitation of the study is the fact that the non-potable water fraction had to be estimated based on expert opinion from the store management, as there were no measurements of the water end use. Still, the sensibility analysis carried out reveals a difference of only 4% in terms of water savings, but representing a difference of roughly €50 per month in financial terms. Taking the lowest non-potable water consumption fraction (70%), this amount represents an increase of 15% in water cost savings. Nevertheless, RWH systems are relevant solutions to achieve important water saving in the retail sector, which would influence retailers’ direct water footprint positively and there is a high probability for the financial viability, particularly if considered during the design stage to avoid the need to duplicate the water distribution network.

Climate change increasingly encourages the search for resources’ conservation solutions, either individually by local promoters or in partnerships with local authorities. In the future, to promote the widespread of RWH systems, the analysis of case studies in other geographic locations could be of relevance. A more detailed estimation of the cost structure for the implementation of RWH systems in new or existing stores is also foreseen in our future research.

## Data Availability

Data supporting reported results can be found at [dataset 1] Ferreira, Ana Sofia (2018), “Combined carbon and energy intensity benchmarks for sustainable retail stores—CSR Reports,” Mendeley Data, v1. DOI: 10.17632/gnygmhvv8d.1 and [dataset 2] Santos Ferreira, Ana Sofia (2020), “Water intensity benchmarks for sustainable retail stores—CSR Reports,” Mendeley Data, V2, doi: 10.17632/gnygmhvv8d.2.

## Appendix

Figures 9 and 10
